# Factors Influencing Variations in Hospitalization for Diabetes with Hypoglycemia

**DOI:** 10.3390/jcm7100367

**Published:** 2018-10-18

**Authors:** Waleed Kattan, Thomas T. H. Wan

**Affiliations:** 1 Department of Health Services and Hospitals Administration, College of Economics and Management, King Abdulaziz University, Jeddah 21589, Saudi Arabia; wmkattan@kau.edu.sa; 2 Department of Health Management and Informatics, University of Central Florida, Orlando, FL 32816, USA

**Keywords:** hypoglycemia, hospital utilization, predictors of hospital length of stay, costs of care, high-risk profile of hospitalized diabetes

## Abstract

Many studies have explored risk factors associated with Hypoglycemia (HG) and examined the variation in healthcare utilization among HG patients. However, most of these studies failed to integrate a comprehensive list of personal risk factors in their investigations. This empirical study employed the Behavioral Model (BM) of health care utilization as a framework to investigate diabetes’ hospitalizations with HG. The national inpatient sample with all non-pregnant adult patients admitted to hospitals’ emergency departments and diagnosed with HG from 2012 to 2014 was used. Personal factors were grouped as predictors of the length of stay and the total charges incurred for hospitalization. High-risk profiles of hospitalized HG patients were identified. The analysis shows the need for care factors are the most influential predictors for lengthy hospitalization. The predisposing factors have a limited influence, while enabling factors influence the variation in hospital total charges. The presence of renal disease and diabetes mellitus (DM) complications played a key role in predicting hospital utilization. Furthermore, age, socio-economic status (SES), and the geographical location of the patients were also found to be vital factors in determining the variability in utilization among HG patients. Findings provide practical applications for targeting the high-risk HG patients for interventions.

## 1. Introduction

Diabetes is a major health problem [1]. Despite many efforts to combat this disease, factors influencing hospital utilization by patients with diabetes are poorly understood. When diagnosed, doctors advise their patients to change their lifestyle through improving nutrition, and maintaining regular physical exercise [2]. The efforts aim to achieve better control of glucose levels in the patients’ blood. However, few patients succeed in controlling their condition with lifestyle changes. Most patients require supplementation with “glucose-lowering medications”, which help in reducing the amount of glucose circulating in their blood to achieve what is known as a state of “glycemic control” [3].

Unfortunately, sometimes the blood glucose level (BGL) falls below an optimal level and patients develop an event called “hypoglycemia” (HG) [4]. Episodes of HG can be mild, moderate, or severe, depending on the drop in blood glucose below the optimum BGL [5]. It is well-known that HG is usually associated with higher utilization of healthcare resources such as emergency department (ED) visits, physician visits, Intensive Care Unit (ICU) transfers, and medications [6]. HG is common among patients with diabetes. It is estimated that 25% of hospital admissions for diabetes are because of HG [7]. Therefore, many studies conducted explore risk factors associated with HG. However, these studies lack a solid theoretical framework to guide the analysis and result in insufficient knowledge of predictors for HG hospitalization.

A 2015 systematic review and meta-analysis of population-based studies revealed the incidence of HG episodes is about 20 times a year per diabetic patient [8]. While not all HG events are considered severe and many can be self-managed, high-costs result when patients seek medical assistance through the ED and frequently lead, to an admission. The Centers for Disease Control and Prevention (CDC) estimated that nearly 300,000 ED visits for adults in the U.S. occurred in 2011, with HG being the first-listed diagnosis and diabetes as another leading diagnosis [9]. Analysis of data from the 1993–2005 National Hospital Ambulatory Medical Care Survey showed there were about five million ED visits due to HG, translating to an ED visit rate of 34 (95% CI 30–37) per 1000 diabetic patients with HG [10].

Hospitalization of ED-treated HG cases is also common. A study published in the Journal of the American Medical Association found almost one-third (29.3%, CI 21.8%–36.8%) of the cases presented to ED with HG resulted in hospitalization [11]. Ginde et al. [10] also found that one-fourth of ED-treated HG cases were admitted to the hospital. There are other studies showing patients with diabetes and HG have higher odds of being hospitalized and readmitted [12,13]. Analysis of the 10 years’ worth of records (1998–2009) from the National Health Insurance Research Database of Taiwan with over 77,600 T2DM patients showed individuals with HG had 3.45 times higher risk of being hospitalized [12]. The utilization of healthcare services seemed to be higher among those with more severe HG. A recently published systematic review and meta-analysis estimated about 10% of serious HG cases required hospital treatment in the ED or admission [13]. Davis et al. [14] conducted a longitudinal observational cohort study with data for 1999–2006 focusing on diabetic patients and found that 24.2% of severe cases requested ambulance only, 43.9% visited the ED, and about 31.8% needed to be admitted to the hospital. A Canadian study with data for 2004–2009 found patients who experienced previous episodes of severe HG had higher risks for hospitalizations than those who never experienced severe episodes (HR 2.80, 95% CI 1.55–5.06) [15]. 

In addition to the increasing odds of ED visits and hospital admissions among patients with HG, those admitted to the hospital tended to stay an average of three days longer than those without HG [16,17,18]. Moreover, they were at a higher risk of being transferred to the Intensive Care Unit [19,20,21]. Readmission rates among patients with diabetes tend to be higher among those who experience HG [22,23]. As previously described, the increase in utilization related to HG results in tremendously higher treatment costs than for those without HG [6,22,23].

There are numerous, well-conducted studies on risk factors for HG as well as hospitalization of HG events. Studies focused on risk factors usually included national data and concentrated on the incidence and prevalence of the HG events. In addition, many of these studies were specific and focused solely on HG risks among certain medications, specific age groups (e.g., the elderly), or specific comorbidities (e.g., kidney disease). These studies provided useful information on relevant risk factors of HG. However, this review highlighted that other studies lack a holistic approach, which integrates risk factors into a coherent predictive model with varying levels or intensities of utilization. Most of these studies did not control confounding factors in the analysis. Additionally, the analysis of risk factors was descriptive in nature or included minimal use of predictive modeling.

The burden that HG imposes on diabetic patients and the overall healthcare system is enormous. At the patient level, diabetics with HG usually have higher out-of-pocket expenditures, greater risk for short- and long-term complications, and higher mortality rates than diabetics who do not have HG. In addition, HG episodes usually require hospitalizations and are linked to longer hospital stays and higher mortality risks [13]. Furthermore, HG has a detrimental impact on the economy and consumption of healthcare resources. Therefore, there is a pressing need for studies that assist in identifying the high-risk profile of subgroups that are likely to have a lengthy hospitalization or to incur more costs of care during hospitalization. 

This study employed Andersen’s Health Behavioral Model of health care utilization (BM) [24] as a framework to examine the problem of HG. This holistic approach identifies different risk factors and their implications for improving patients’ utilization. In turn, this study provides rich information to healthcare providers for understanding how to target a high-risk patient population for intervention and reduce the occurrence of HG-related hospitalization.

Theoretical Framework. One of the most widely acknowledged models that is used as an explanatory framework in identifying predictors of health care utilization is the BM, which was developed in the 1960s by Ronald M. Andersen [25,26,27,28,29,30,31]. The BM is a multivariate model that integrates three different individual and social determinants of health services utilization: Predisposing (P), Enabling (E), and Need-for-care (N) component. 

Predisposing component: Personal characteristics may predispose an individual to specific episodes of illness [28]. The propensity to utilize medical services differs from one patient to another, and is subject to a variety of social and demographic factors [31]. Based on the previous studies that examined risk factors of HG, this study included the following most commonly used risk factors under predisposing factors: age, gender, and ethnicity [32]. In addition, dementia, depression, tobacco use, alcohol use, and drug abuse were included. These biosocial determinants were included to be consistent with other studies that employed the BM [24].

Enabling component: For people who have the predisposing component, there are means that enhance or inhibit their use of health services [28,31]. This study considered the most relevant enabling factors related to HG hospitalization such as medical insurance type and socio-economic status [32,33]. The geographical location of the patient and the hospital were also included since they historically show a strong influence on utilization and outcome among HG patients.

Need-for-care component: This refers to the level of illness that the individual has [14]. Parallel to the presence of predisposing and enabling conditions, illness is required to be perceived by the patient or his family for the patient to proceed and use health services, such as a clinic or hospital visit [28,33]. Among all other factors, the need for care is considered the most immediate cause of health service use. While there are plenty of elements that can be included under the (N) component, this study included a group of factors that were commonly found to impact service utilization among HG patients, such as DM complications, type of DM, BMI underweight, hypertension, liver failure, kidney disease, uncontrolled DM (A1C > 7%), and the Charlson Comorbidity Index (CCI).

Utilization: The two variables used are the length of stay (LOS) of patients in an acute care hospital and total charges (cost).

The two-fold purpose of this study was: (1) to identify the relative importance of the determinants in predicting LOS and cost/charge of hospitalization; and (2) to identify a high-risk profile of HG patients who are likely to experience longer LOS and higher costs during their hospitalization.

Research Questions. The three research questions considered are as follows:

1. When other factors are held constant, what is the profile of hospitalized diabetics with HG in experiencing longer LOS?
2. When other factors are held constant, what is the profile of hospitalized diabetics with HG in incurring higher total charges?
3. When the effects of other predictors are simultaneously considered, is the need-for-care or total charges the most important predictor of LOS?

## 2. Materials and Methods

### 2.1. Design

A non-experimental and retrospective design was employed in this study by utilizing inpatient data from a national database for years 2012, 2013, and 2014 from 12 states in the United States. 

### 2.2. Data Source and Sample

The study patients were based on the Healthcare Cost and Utilization Project (HCUP), a national database in the United States. HCUP is a group of healthcare databases sponsored by the Agency for Healthcare Research and Quality (AHRQ) [34]. HCUP databases aggregates different data collection efforts of State and Federal organizations, hospital associations, and private data organizations, to establish a unified national information resource of encounter-level health care data [35]. Since 1988, data were gathered every year from 47 States and the District of Columbia, representing 97% of all inpatient hospital discharges. AHRQ converts administrative health care data that were acquired from HCUP partners into uniform databases that are relatively easy to use by researchers.

The data were extracted from the National Inpatient Sample (NIS), a compendium of State inpatient databases in HCUP. As approximated by the NIS, there is a 20% stratified sample of all discharges from community hospitals, not including rehabilitation or long-term acute care hospitals [34]. The data include all patients, regardless of payer, including individuals covered by Medicaid, Medicare, private insurance, or uninsured. This is considered the largest all-payer inpatient care database in the U.S. including data from more than seven million hospital stays each year [35]. 

This study included all cases presented to the ED in the years 2012, 2013, and 2104. Based on the International Classification of Diseases Ninth Revision (ICD-9) diagnostic codes, only adults 18 and older, who were diagnosed with T1DM or T2DM and were diagnosed with HG were included in the study. The unit of analysis of this study was individual patients. Because of the nature of the study, non-diabetics and diabetics without HG were excluded from the study. Patients who were younger than 18 years were also excluded because genetic diseases are the predominant factors leading to HG among children [36]. In addition, factors that lead to HG are different among newborns, infants, toddlers, and older children [37]. Lastly, when compared to non-pregnant adults, HG during pregnancy has different maternal risk factors, different pregnancy outcomes, and a wide-range of fetal-related complications [38,39]. Therefore, all pregnant women were excluded from this study.

### 2.3. Study Variables

The BM is the principal framework for this study. Different indicators or variables were considered under each of the main three constructs/components: predisposing, enabling and need-for-care factors. In addition, the study also included other indicators under the utilization construct. Table 1 summarizes the study variables.

Predisposing component (P): As mentioned earlier, the study includes the main key variables used in previous studies that were found to be relevant risk factors related to HG among diabetic patients. The indicators for the predisposing component or risk propensity profile in this study include: age, ethnicity, gender, dementia, depression, and healthy life. The age of the patients was collected in years. Gender was either male or female. Ethnicity was African-American, Hispanic, or others. Based on evidence from the literature review, these categorizations showed that African Americans and Hispanics had higher odds of developing HG when compared to others. Regarding healthy life status, patients were categorized into three groups: those who do not drink, smoke, or abuse drugs; those who do one of these; and those who do two or more.

Enabling component (E): The study included the four major indicators for the enabling construct which were frequently used in previous studies to facilitate or impede the use of health services: medical insurance type, socioeconomic status (SES) level, the location of the patient, and the hospital location. Health insurance was categorized into two groups: those who were on Medicaid and those who had Medicaid, private insurance, or no insurance. This categorization is relevant to this study as Medicaid patients were found to be the sickest group when compared to the other groups [40]. Regarding SES, the categorization was based on the median household income for the patient. Of the four categories that represent income percentile level, category one represented the poorest from the 0–25th percentile, while category four represented the richest from the 75th to 100th percentile. Hospital locations were categorized into urban or rural. Lastly, patient locations were categorized into five groups based on the population density of the county related to patients’ homes: the smaller the category number, the smaller the county population size.

Need for Care (N): Based on the review of the literature, the indicators for N were the T2DM the presence of diabetes-related complications (DM_complications), uncontrolled diabetes (uncontrolled_DM), underweight (BMI underweight), hypertension, hyperlipidemia, liver disease (liver_dis), renal disease (renal_disease), and cancer. All of these variables are dichotomous in nature: No = 0, Yes = 1. These variables were extracted from the ICD-9 codes associated with each case in the database. As discussed earlier, CCI was found to have a broad range of consequences in addition to HG, which included cardiovascular complications, falls, higher costs, longer LOS, and more risks to mortality. Therefore, it was more appropriate for this study to consider CCI as a control variable. It is also worth mentioning that this approach of controlling CCI was employed in numerous previous studies that examined risk factors for HG among diabetic patients [13,41,42,43,44,45].

Utilization (U): As discussed earlier, the two utilization variables (dependent variables) examined in this study are LOS and the total charges. Total cost refers to the total charges of care in U.S. dollars. The number of hospital days does not necessarily correlate with the total cost of the hospital stay. In addition, some variables might influence LOS or the total charges without affecting the other variables. Therefore, the study treated the two utilization variables separately.

### 2.4. Statistical Analysis

By utilizing descriptive statistics, this study examined general characteristics of the data and any missing results, using descriptive statistics. IBM Statistical Package for Social Sciences (SPSS version 21) was used to look for multicollinearity or correlations among different variables. Highly correlated variables (>0.9) were not included into the multivariate statistical model because of the threat of multi-collinearity problems.

Decision Tree Regression (DTREG) is a robust application that is usually used for data mining. This is a process of extracting relevant information from the database, to generate user-friendly and easy-to-interpret models such as the decision tree model [46]. The software generates a series of branched “nodes” or mutually exclusive subgroups that describe the relationship between predictor variables and utilization variables. Only statistically significant predictor variables that explained some variance in each dependent variable were shown in making the splits of subgroups. The relative importance of each statistically significant predictor variable is graphically presented. By examining the mean and standard deviation values in each terminal node or final subgroup (no more splits occurred), we can identify higher LOS or cost groups as high-risk subgroups for developing group-specific interventions [47]. 

## 3. Results

### 3.1. Descriptive Analysis

After merging the databases for the three years, cases involving patients that were younger than 18 years were excluded. All cases that included pregnant women were also excluded. Only cases that were admitted through ED, diagnosed with T1DM or T2DM, and presence of HG were selected. When examining the selected cases, the variables (sex, AA_Hisp, patient_loc, Medicaid, and SES) had missing data, and the missing data for each of the variables constituted less than 5%. Therefore, missing data was replaced by the calculated mean. When checking for normality, there were few outliers for the variables LOS and total charge, and these outliers were less than 0.3% of the data. Therefore, these outliers were excluded from the analysis to have normally distributed data. Table 2 shows the descriptive statistics of the variables after cleaning the data.

As shown in Table 2, the total sample size was 4822 with no missing data. A sample size of 4822 participants seems sufficient as the general guidelines for normality, the skewness and kurtosis indices, for each variable were inspected to determine its normality. Extreme values were considered when the absolute values for skewness are >3.0, and >10.0 for kurtosis [48]. In examining the results, it seems that the skewness and kurtosis indices for all variables were within the recommended range, except for the three variables under_wt, liver_dis, and cancer, where the values were above the suggested range. The frequency analysis showed that those three variables (under_wt, liver_dis, and cancer) had very skewed distributions, about 10% with a value = 1 vs. 90% = 0. Therefore, these variables were excluded from multivariate analysis.

### 3.2. Correlation Analysis

Since most of the variables in the data were categorical in nature, the Spearman’s rank-order correlation was used to examine the correlation among the variables [49]. The relationship between two variables was expressed as a range from −1 to +1. Correlation coefficients of >0.85 are considered strong positive relationships and <−0.8 are regarded as a strong negative correlation between two variables and may indicate potential problems [48,50]. None of the variables were highly correlated with the other, and therefore all variables were considered in the multivariate analysis.

### 3.3. Decision Tree Regression (DTREG)

As mentioned earlier, DTREG modeling was conducted on each target or dependent variable. Initially, only those predictor variables under (P, E, and N) that were found statistically significant were analyzed separately against the target variable. Afterward, an overall decision tree analysis was conducted, which included high-impact variables on the target variable. Results of the analysis, as well as the decision tree models, are shown following the analysis of each of the dependent variable.

DTREG for LOS:

As described above, a separate DTREG analysis was conducted for each of the groups of predictor variables influencing LOS. The results of the analysis for the predisposing factor group including age, dementia, no_depression, and HLS resulted in age (relative importance = 100) being the most important variable that influenced LOS followed by HLS, no_depression, and dementia (relative importance = 18.6, 12.4, 9.5, respectively). For the enabling factor group, SES was of highest importance followed by patient_loc and then urban_hosp (relative importance = 100, 72.1, 63.3, respectively). Regarding the need-for-care factor group, the top two predictor variables were DM_comp and renal_disease (relative importance = 100, 62.5, respectively), followed by hyperlipidemia, DMII, and hypertension (relative importance = 49.4, 47.15, 45.29, respectively).

**Figure 1:**
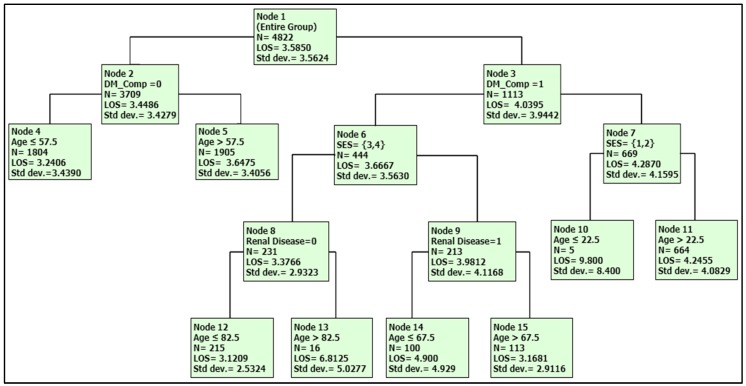
DTREG tree of the predictors for LOS.

The final DTREG analysis for LOS included the following statistically significant predictor variables: age, SES, DM_comp, renal_disease hyperlipidemia, T2DM, and hypertension. The results of the analysis revealed that, among all factors, age had the highest relative impact on LOS, followed by SES, DM_comp, renal_disease, and hypertension. The proportion of variance explained by the model (*R*^2^) was 7.3%, and RMSE was 3.4. A DTREG tree of the predictors for LOS was generated as well as a summary table for the results, as shown in Figure 1.

The terminal groups for LOS with the average days include Node 12 (3.1 days), Node 4 (3.2), Node 5 (3.2), Node 15 (3.2), Node 11 (4.2), Node 14 (4.9), Node 13 (6.8), and Node 10 (9.8). Two highest LOS subgroups are: (1) Node 10 characterized as diabetics with the complication, lower SES, and younger than 23 years of age; and (2) Node 13 characterized as diabetes with the complication, higher SES, without renal disease, and older than 82 years of age (Table 3 and Figure 1).

**Figure 2:**
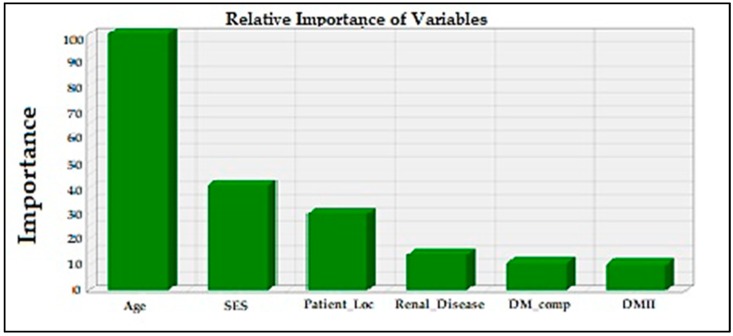
Relative importance of statistically significant variables in explaining the variance in the overall LOS.

Of the seven predictors of LOS with varying explanatory power shown in Figure 2, age appeared to be the most influential variable, followed by SES, DM complication, renal disease, hypertension, hyperlipidemia and DMII. 

DTREG for Total Charge:

Similar steps were conducted for the DTREG analysis with the total charge. Again, results of the analysis for the (P) group showed age (relative importance = 100) is by far the most important variable that influenced total charge followed by HLS, no_depression, and dementia (relative importance = 15.2, 11.2, 7.1, respectively). For the (E) group, patient_loc was the most important, followed by SES and then urban_hosp (relative importance = 100, 23.7, 13.4, respectively). Regarding the (N) group, the top two predictor variables were renal_disease and DM_comp (relative importance = 100, 49.3, respectively), followed by DM II, hyperlipidemia, and hypertension (relative importance = 27.5, 19.9, 18.6, respectively).

**Figure 3:**
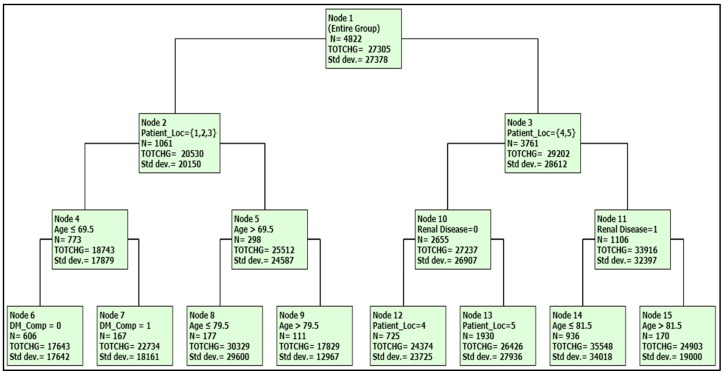
DTREG tree of the predictors for the total charge.

The final DTREG analysis included the following statistically significant predictor variables: age, patient_loc, SES, urban_hosp, renal_disease, DM_comp, and T2DM. The results of the analysis indicated that age had the highest relative impact on total charge, followed by patient_loc, SES, renal_disease, DM_comp, and T2DM. The variance in total charge explained by the model (*R*^2^) was 11.97%, and RMSE was 25.687. A DTREG tree of the predictors for total charge is presented in Figure 3.

As shown in Figure 2 and Table 4, highest charges were associated with those younger than 81.5 years who are located in larger metropolitan areas and who had renal disease ($33,548), followed by those who were 70–80 years old and who lived in smaller towns ($30,329). In contrast, those who were younger than 70, had no DM complication and lived in smaller towns had the lowest charges (Node 6), followed by those elderly DM patients who were older than 80 and lived in smaller towns (Node 9). 

**Figure 4:**
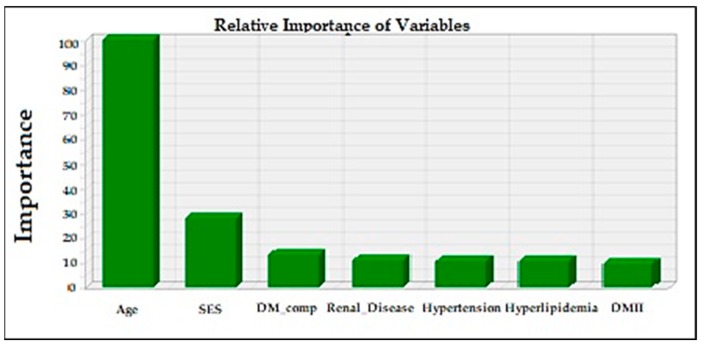
Relative importance of statistically. significant predictor variables in explaining the variation in total charge.

Of the six predictors of total charge with varying explanatory power shown in Figure 4, age accounted for the most variance, followed by patient location, SES, renal disease, DM complication, and DMII.

## 4. Discussion

This study attempted to answer three research questions pertaining to the high-risk profiles of patients with diabetes for longer LOS or higher total charge for hospitalization. Furthermore, the study examined the relative influence of predictor variables in understanding the variability in hospitalization of HG patients. The findings provided strong evidence in portraying the high-risk profiles of user groups analyzed by LOS and total charge independently. 

For LOS as a dependent variable, age, patients located in metropolitan areas, and medical need factors such as the presence of complication(s), renal disease, hypertension, and DM I appeared to influence the duration of hospital stay. It is important to note that specific targeted interventions should be designed to address avoidable hospitalizations for diabetes. The one-size-fits-all strategies are inappropriate for patient groups with varying degrees of medical need. The preventive practice to identify diabetes complication(s) and poly chronic conditions is essential for developing care management modalities that may enhance self-efficacy or adherence to medical regimens [51].

For the total charge variable-age, metropolitan size, SES and medical care needs (e.g., renal disease, DM complication(s), and T1DM) are relevant predictors of having incurred a higher total hospitalization charge. Thus, patient-specific strategies could be devised to identify and adopt value-added services to replace expensive hospitalization. Similar to LOS, preventive strategies for reducing avoidable hospitalization (i.e., readmission) require more thorough exploration in clinical research so that cost-effective self-care practices among diabetics could be developed and used. One viable approach is to search for mechanisms and avenues to coordinate or integrate community-based care with acute care for patients who are suffering poly chronic conditions [51].

The results of the study also revealed the need for care factors (N) are an important predictor for LOS and total charge. By observing the high-risk groups for longer LOS and higher costs of care, practitioners may find feasible solutions to curtail expensive hospitalizations as demonstrated by practitioners in a community and coordinated care network for potentially expensive patients with poly chronic conditions in Camden, New Jersey. Results of this study also showed that the geographical location of diabetic patients with HG impacts the variation in total charge of hospitalization. Recent geographical distribution of diabetes data in Florida shows 14% of adults in urban areas have diabetes, compared to 15.5% in suburban areas and 18.7% in rural areas [52]. A recent study conducted in eight southeastern states revealed racial/ethnic disparities in diabetes hospitalization of rural Medicare beneficiaries [53]. Therefore, this study shows there is a need for interventions tailored to residents in rural areas. These measures might include conducting community needs assessments to evaluate the availability of healthcare services in patients’ neighborhoods as well as examining other psycho-social challenges present in their environment that might affect their diabetes-related care.

With recent transition toward the value-based payments (VBP), healthcare providers are encouraged to deliver more high-quality services at a lower cost [54]. This is due to reimbursement based on quality rather than quantity of visits or the number of services provided [55]. In addition, stakeholders impose continuous pressure on healthcare organizations to provide sustainable, high quality, and cost-effective services [56]. Therefore, understanding groups at high-risk for developing complications or using more services for a common disease, such as DM, is crucial for healthcare providers [57].

This study revealed certain groups are at higher risk of longer LOS and total charges. Hospitals can employ a digital predictive model to identify high-risk patients and proactively provide the best care for them. In addition, healthcare providers can target this group with educational interventions by employing the Knowledge, Motivation, Attitude, Preventive Practice, and Outcomes (KMAP-O) framework to improve their health status, prevent severe adverse outcomes, and reduce their level of healthcare utilization [58,59]. Last, collaborations among different levels of providers (e.g., clinics, hospitals, long-term, and post-acute care organizations) might also facilitate monitoring wearable devices among high-risk DM patients to prevent HG episodes [60].

Although this is a robust study with more than 4000 patients from the national database, there are certain limitations. First, the study used secondary data, which is the largest national database available. However, the nature of secondary data usually limits the variables to those existing in the database. Therefore, identified variables, (e.g., patients’ educational attainment, the presence of a primary care physician, duration of DM, history of previous HG, medications used, and whether the patient was transferred to ICU) would be important when examining HG risk factors, but they were not recorded in the database. In addition, variables that were already included in the database need further clarification to be useful. For instance, the data did not differentiate between those who were admitted with HG from those who developed HG after admission. Another issue is that, because the database included only inpatient data, only short-term in-hospital complications were captured. Thus, long-term complications, such as readmissions and mortality after discharge could not be captured. Moreover, other HG patients who sought medical assistance through clinics or other avenues are missing.

There were assumptions related to the data used in this study. The first assumption was that any diagnosis not recorded in the patients’ file with ICD codes was considered to be absent. However, because of the nature of the ICD coding and its sole purpose for medical billing, there were some occasions in which some problems happened to patients and might have been recorded in the doctors’ notes, but they were not recorded under the patients’ ICD codes because the hospital would not have been reimbursed for them. These missing pieces of information lead to a second assumption about the accuracy of the data: since there was no way to check the accuracy of data entry into the database, the study assumed that all data are accurate. The last assumption relates to the variables. For example, the study assumed all patients had one source of insurance coverage, as the data recorded only one type of insurance provider for each patient. However, there are some dually eligible patients with both Medicaid and Medicare. Unfortunately, information about patients with dual insurance could not be attained, so the assumption made for this study was that all patients had only one single insurance provider.

Future research should formulate a prospective study to allow the inclusion of other key variables such as the duration of DM [61,62], type of glucose-lowering medications [63,64,65,66,67], type of antibiotics [68,69] and transfer to ICU [70,71] to explore their impact on HG.

In addition, it is recommended that further research be conducted concerning the timing and frequency of HG episodes among admitted patients as well as separating those who come to ED with HG from others who develop in-hospital HG. Another area of possible future research would be to explore the long-term HG effects such as readmissions [14,72], history of HG, and annual frequency of HG [73,74], as well as other complications that are treated in outpatient settings. Lastly, HG can occur alongside diseases other than diabetes. It might be important to conduct studies that examine HG among patients without diabetes and their risk factors, utilization, cost, and outcomes to compare that group to patients with diabetes. 

## 5. Conclusions

To the best of our knowledge, the present study is the first aimed at examining the multiple factors that contribute to HG and their effects on LOS and total charge while employing Andersen’s behavioral systems framework. The use of the largest national inpatient database combined with the robust analytical methods provided rich information about HG hospitalization. By conducting predictor tree analysis of risk factors, the study further explored the probability of the various variables influencing the variability in LOS and total charge, which provided a better picture about HG risk factors and their individual impacts on each utilization variable. Thus, especially with the current shift toward value-based payment, these results provide practical applications for healthcare providers as they could target the high-risk HG patients for avoiding hospitalization or rehospitalization among diabetic patients. Based on the study findings, healthcare providers can more easily proactively deal with the high-risk groups who are elderly patients with: comorbidities, diabetes-related complications, and who present with renal disease. Targeting those groups could help reduce the frequency and severity of HG episodes. Consequently, better results could be achieved, such as shorter LOS and lower cost. 

The study results also have great policy implications for decision makers regarding how to approach the growing problem of DM in metropolitan areas. One major finding is the pronounced impact of age on LOS and total charge. With the increasing number of older people and diabetes incidence, policy makers need to integrate innovative policies with collaboration among different stakeholders and experts to enhance patients’ levels of self-care and adherence to recommended care. 

## Figures and Tables

**Table 1:** Operational definition and measurement instruments for study variables.

	Variable Name	Variable Type	Definition	Scale
Predisposing Component (P)				
1	Age	Exogenous	Age of the patient. All adults 18 years and above	Continuous in years
2	Gender	Exogenous	Male or Female	Categorical (Dichotomous): Male = 0; Female = 1
3	AA_Hisp	Exogenous	The ethnicity of the patient. Whether patient’s ethnicity is African American or Hispanic or not	Categorical: 1 = African American or Hispanic; 0 = Others
4	Dementia	Exogenous	Patient has dementia or not	Categorical (Dichotomous): No dementia = 0; Yes = 1
5	No Depression	Exogenous	Patient has no depression	Categorical (Dichotomous) No depression = 1; Depression = 0
6	Healthy lifestyle (HLS)	Exogenous	Refer to the healthy lifestyle for the patient, which is tobacco-free, alcohol-free, and no drug abuse	Categorical: 2 = Patient has a healthy lifestyle with no smoking, no alcohol, and no drugs; 1 = Patient does one of the above; 0 = Patient does 2 or more of the above.
Enabling Component (E)				
1	Medicaid	Exogenous	Whether the patient is covered by Medicaid or not	Categorical (Dichotomous): Medicaid = 1; Others (Medicare, Private, no insurance) = 0
2	SES	Exogenous	The socio-economic status of the patient based on the median household income for patient	Categorical: 1 = 0–25th percentile; 2 = 26th to 50th percentile; 3 = 51st to 75th percentile; 4 = 76th to 100th percentile
3	Urban_hosp	Exogenous	Hospital located in an Urban or rural area	Categorical (Dichotomous): Urban = 1; Rural = 0
4	Patient Location	Exogenous	Patient home location. Based on the location’s county population.	Categorical: 1 = Not metropolitan or micropolitan county; 2 = Micropolitan county; 3 = Counties in metro areas of 50,000–249,999 population; 4 = Counties in metro areas of 250,000–999,999 population; 5 = Counties of metro areas of ≥1 million population
Need Component (N)				
1	DM Complications	Exogenous	Patient has any DM specific complication (eye, neurological, cardiac, renal, others)	Categorical (Dichotomous): No complications = 0; Yes = 1
2	Uncontrolled DM	Exogenous	Patient has DM that is uncontrolled	Categorical (Dichotomous): No = 0; Yes =1 (AIC > 7.0%)
3	DMII	Exogenous	Patient is T2DM	Categorical (Dichotomous): T2DM = 1; T1DM = 0
4	BMI underweight	Exogenous	Patient BMI category is underweight	Categorical (Dichotomous): No = 0; Yes = 1
5	Hypertension	Exogenous	Patient has hypertension	Categorical (Dichotomous): No = 0; Yes = 1
6	Hyperlipidemia	Exogenous	Patient has hyperlipidemia	Categorical (Dichotomous): No = 0; Yes = 1
7	Liver_dis	Exogenous	Patient has moderate to severe liver disease	Categorical (Dichotomous): No = 0; Yes = 1
8	Renal_disease	Exogenous	Patient has Renal disease	Categorical (Dichotomous): No = 0; Yes = 1
9	Cancer	Exogenous	Patient has malignancy	Categorical (Dichotomous): No = 0; Yes = 1
10	Charlson Comorbidity Index (CCI)	Control	Score of the CCI	Categorical. Scores 1–25
Utilization (U)				
1	Hospital LOS	Endogenous	Patient days in the hospital	Continuous: in days
2	Cost	Endogenous	Total charges in US$ for the admission	Continuous: in USD

AA_Hisp = African American or Hispanic; SES = socioeconomic status; hosp = hospital; DM= diabetes mellitus; DMI or T1DM = Type 1 diabetes; DMII or T2DM = Type 2 diabetes; BMI = body mass index; dis = disease; LOS = length of hospital stay.

**Table 2:** Descriptive statistics.

	Valid	Missing	Mean	Std. Deviation	Skewness	Kurtosis	Range	Min.	Max.	Sum
Age	4822	0	58.42	17.08	−0.22	−0.64	72	18	90	281,700
Sex	4822	0	0.48	0.50	0.08	−1.99	1	0	1	2317
AA_Hisp	4822	0	0.32	0.47	0.78	−1.40	1	0	1	1540
Dementia	4822	0	0.08	0.27	2.94	7.88	1	0	1	377
No_Depression	4822	0	0.61	0.49	−0.44	−1.81	1	0	1	2928
HLS	4822	0	1.57	0.68	−1.31	0.34	2	0	2	7592
Urban_hosp	4822	0	0.77	0.42	−1.30	−0.31	1	0	1	3726
Patient_Loc	4822	0	4.18	1.16	−1.30	0.59	4	1	5	20,140
SES	4822	0	2.21	1.07	0.36	−1.14	3	1	4	10,678
Medicaid	4822	0	0.19	0.39	1.57	0.46	1	0	1	924
CCI	4822	0	2.43	2.43	1.36	2.22	16	0	16	11,715
DM_comp	4822	0	0.23	0.42	1.28	−0.37	1	0	1	1113
Uncont DM	4822	0	0.24	0.42	1.24	−0.45	1	0	1	1138
DMII	4822	0	0.85	0.35	−2.01	2.04	1	0	1	4120
Under_Wt	4822	0	0.14	0.34	3.84	8.53	1	0	1	476
Hyper-lipidemia	4822	0	0.40	0.49	0.39	−1.85	1	0	1	1947
Renal_Disease	4822	0	0.29	0.45	0.95	−1.10	1	0	1	1380
Liver_dis	4822	0	0.06	0.23	3.82	12.61	1	0	1	275
Hyper-tension	4822	0	0.76	0.43	−1.24	−0.47	1	0	1	3679
Cancer	4822	0	0.08	0.27	3.13	7.82	1	0	1	379
LoS	4822	0	3.59	3.56	2.71	9.97	27	0	27	17,287
TOTCHG	4822	0	27,305	27,381	2.75	9.86	205,786	1713	207,499	131,662,579
Outcome	4822	0	0.90	0.98	0.30	−1.70	3	0	3	4336
YEAR	4822	0	2013	0.82	0.00	−1.50	2	2012	2014	9,706,689

TOTCHG = total hospital charge; YEAR = year of the hospital data studied.

**Table 3:** Summary of DTREG analysis of the predictors for LOS—ranked from highest to lowest Subgroups.

LOS/ Node	Characteristics
9.80 (Node 10)	Age < 22.5, with DM complications, SES 1,2
6.80 (Node 13)	Age > 82.5, with DM complications, no renal disease, SES 3,4
4.90 (Node 14)	Age < 67.5, with DM complications and Renal disease, SES 3,4
4.24 (Node 11)	Age > 22.5, with DM complications, SES 1,2
3.64 (Node 5)	Age > 57.5 with no DM complication
3.24 (Node 4)	Age < 57.5 with no DM complication
3.17 (Node 15)	Age > 67.5, with DM complications and Renal disease, SES 3,4
3.12 (Node 12)	Age < 82.5, with DM complications, no renal disease, SES 3,4

**Table 4:** Summary of DTREG analysis of the predictors for total charge—ranked from highest to lowest.

Total Charge ($)/Node	Characteristics
35,548 (Node 14)	Age < 81.5 with renal disease, Patient location 4, 5
30,329 (Node 8)	Age 70–80, Patient location 1, 2, 3
28,426 (Node 13	Patient location 5 and no Renal disease
24,933 (Node 15)	Age > 81.5 with renal disease, Patient location 4, 5
24,074 (Node 12)	Patient location 4 and no Renal disease
22,734 (Node 7)	Age < 70, with DM comp, Patient location 1, 2, 3
17,829 (Node 9)	Age > 80, Patient location 1, 2, 3
17,643 (Node 6)	Age < 70, no DM comp, Patient location 1, 2, 3
